# Genome-wide association and transcriptional studies reveal novel genes for unsaturated fatty acid synthesis in a panel of soybean accessions

**DOI:** 10.1186/s12864-019-5449-z

**Published:** 2019-01-21

**Authors:** Xue Zhao, Haipeng Jiang, Lei Feng, Yingfan Qu, Weili Teng, Lijuan Qiu, Hongkun Zheng, Yingpeng Han, Wenbin Li

**Affiliations:** 1 Key Laboratory of Soybean Biology in Chinese Ministry of Education (Key Laboratory of Soybean Biology and Breeding/Genetics of Chinese Agriculture Ministry), Northeast Agricultural University, 150030, Harbin, China; 2 Institute of Crop Science, National Key Facility for Crop Gene Resources and Genetic Improvement (NFCRI) Chinese Academy of Agricultural Sciences, Beijing, 100081 China; 3 Bioinformatics Division, Biomarker Technologies Corporation, Beijing, 101300 China

**Keywords:** Soybean, Unsaturated fatty acids, SNPs, Genome-wide association studies, Candidate genes, Expression profile

## Abstract

**Background:**

The nutritional value of soybean oil is largely influenced by the proportions of unsaturated fatty acids (FAs), including oleic acid (OA, 18:1), linoleic acid (LLA, 18:2), and linolenic acid (LNA, 18:3). Genome-wide association (GWAS) studies along with gene expression studies in soybean [*Glycine max (L.)* Merr.] were leveraged to dissect the genetics of unsaturated FAs.

**Results:**

A association panel of 194 diverse soybean accessions were phenotyped in 2013, 2014 and 2015 to identify Single Nucleotide Polymorphisms (SNPs) associated with OA, LLA, and LNA content, and determine putative candidate genes responsible for regulating unsaturated FAs composition. 149 SNPs that represented 73 genomic regions were found to be associated with the unsaturated FA contents in soybean seeds according to the results of GWAS. Twelve novel genes were predicted to be involved in unsaturated FA synthesis in soybean. The relationship between expression pattern of the candidate genes and the accumulation of unsaturated FAs revealed that multiple genes might be involved in unsaturated FAs regulation simultaneously but work in very different ways: *Glyma.07G046200* and *Glyma.20G245500* promote the OA accumulation in soybean seed in all the tested accessions; *Glyma.13G68600* and *Glyma.16G200200* promote the OA accumulation only in high OA germplasms; *Glyma.07G151300* promotes OA accumulation in higher OA germplasms and suppresses that in lower OA germplasms; *Glyma.16G003500* has the effect of increasing LLA accumulation in higher LA germplasms; *Glyma.07G254500* suppresses the accumulation of LNA in lower OA germplasms; *Glyma.14G194300* might be involved in the accumulation of LNA content in lower LNA germplasms.

**Conclusions:**

The beneficial alleles and candidate genes identified might be valuable for improving marker-assisted breeding efficiency and exploring the molecular mechanisms underlying unsaturated fatty acid of soybean.

**Electronic supplementary material:**

The online version of this article (10.1186/s12864-019-5449-z) contains supplementary material, which is available to authorized users.

## Background

Soybean (*Glycine max* L. Merr.), a major oil crop worldwide, contributes 31% of the global supply of edible vegetable oil [1, 2]. Soybean oil consists of saturated fatty acids (FAs), including palmitic acid (PA, 16:0) and stearic acid (SA, 18:0) and unsaturated FAs such as oleic acid (OA, 18:1), linoleic acid (LLA, 18:2), and linolenic acid (LNA, 18:3). The amounts and relative proportions of the five FAs, especially unsaturated FAs directly determine the flavor, stability, and nutritional value of soybean oil [3]. Consumption of soybean oils with higher OA levels is desirable because this monounsaturated FA could improve storage time, which increases the price of the soybean oil and in the cost of generating unhealthy trans fat for humans [4]. LLA and LNA can’t be synthesized by the human body, and in turn have to be obtained from the daily diet [5]. Although the nutritional value of unsaturated FAs benefits humans, the high levels of LLA and LNA in soybean oil results in low oxidative stability and rapid rancidity, as well as reduces storage time of soybean oil [6]. Hence, the increase in OA and decrease in LLA and LNA contents could improve the quality of soybean oil for human consumption and thus has become an important goal of soybean breeders.

The unsaturated FAs in soybean are typical quantitative traits controlled by major and minor genes/quantitative trait loci (QTLs) and are easily affected by environment or by genotype X environment interaction factors [7]. Identifying unsaturated FA QTLs based on biparental mapping populations through linkage mapping accelerates the development of soybean lines with reasonable unsaturated FA content and relative proportions to meet the widespread demand for soybean oil. Currently, nearly 200 QTLs associated with unsaturated FA concentrations have been reported (http://www.soybase.org), which distributed across 20 chromosomes (Chr.) or linkage groups (LG) of soybean. However, the utilization in breeding programs of these QTLs for developing soybean lines with reasonable unsaturated FA content is limited due to poor resolution and accuracy of these QTLs, which is caused by lower recombination events based on biparental mapping populations or genomic regions with higher linkage disequilibrium (LD) [8]. Another reason for the reported lower utilization efficiency of these QTLs is lower consistency across different genetic backgrounds. Unlike linkage analysis, genome-wide association studies (GWAS) have been utilized as an alternative method to identify unsaturated FA QTLs [8]. GWAS based on the natural population have more extensive recombination events, thereby resulting in shorter LD segments and therefore increased resolution and accuracy of marker-phenotype associations. The developments in genome sequencing and single nucleotide polymorphism (SNP) genotyping technology have promoted the applicability of GWAS in soybean, which has been used to determine genomic regions that are associated with resistance to biological stress, including soybean cyst nematode [9–11] and white mold [12], abiotic stress [13, 14], yield-related traits, including seed weight [15], flower time [16], and seed composition such as seed oil content [17] and seed protein content [18]. For unsaturated FAs, presently only two GWAS based on the high-throughput technologies have been reported. A targeted GWAS was used to analyze the genetic architecture of unsaturated FAs using only 1536 SNPs and 421 diverse accessions, and the results showed that 8, 12, and 5 Quantitative Trait Nucleotide (QTNs) were associated with LLA, LNA, and OA content [19]. Compared to previously identified known genes/QTLs, only a few novel significant association signals were detected due to the utilization of only thousands of markers [19]. Leamy et al. [20] conducted a GWAS of unsaturated FAs using nearly 30,000 SNPs and found 9, 5, and 5 QTNs were associated with LLA, LNA, and OA content. The results of Leamy et al. [20] did not fully agree with those of other studies because different cultivated soybeans were used as parents in each linkage analysis. However, by late 2017, no studies have been conducted to identify QTNs and candidate genes underlying unsaturated FA content of cultivated soybean based on enormous SNP markers phenotype evaluation in multi-environments.

The present study performed a GWAS of unsaturated FAs based on 36,981 SNPs and 194 germplasms from Northeastern China. The aim of the present study was to identify QTNs associated with unsaturated FA levels, to screen candidate genes located within SNP regions, and to verify the role of candidate genes related to soybean FA metabolism.

## Results

### Phenotyping and statistical analysis for seed unsaturated FA content

**Fig. 1:**
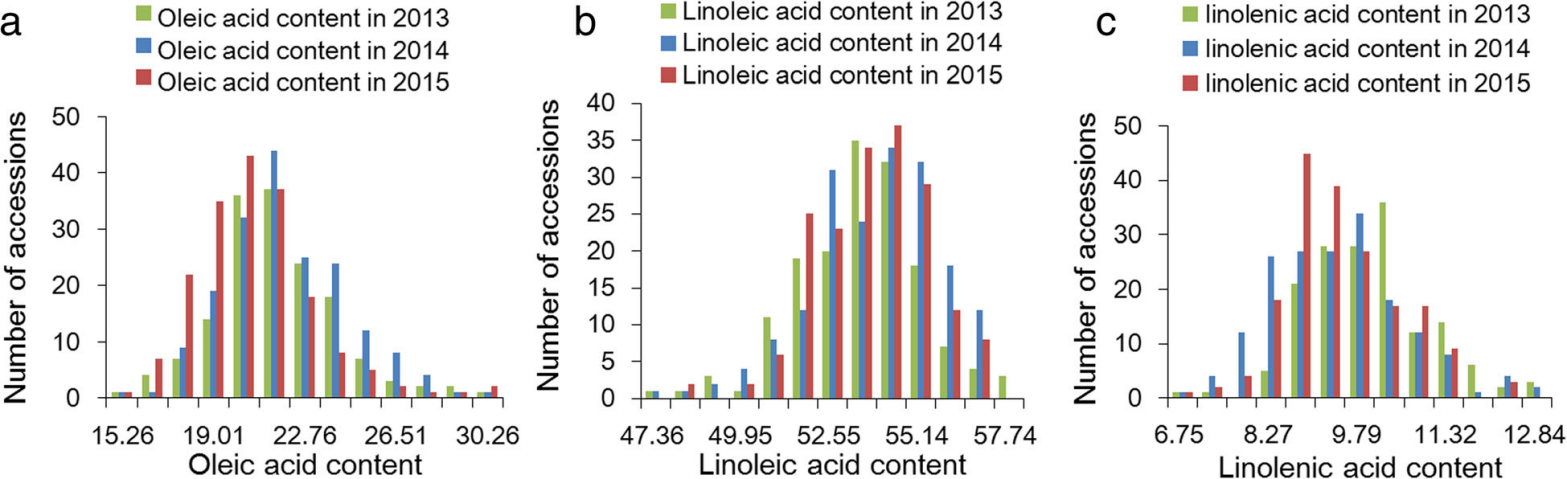
Phenotypic variation in unsaturated fatty acid content in soybean seeds of the tested accessions. **a**: oleic acid content, **b**: linoleic acid content, **c**: linolenic acid content

There was a wide range of phenotypic variations in the levels of the three unsaturated FAs, particularly those of OA in 194 accessions (Fig. 1 and Additional file 1: Table S1). Genetic parameters, including mean, standard deviation, coefficient of variation, skewness, and kurtosis for OA, LLA, and LNA content in the tested GWAS panel were calculated (Additional file 1: Table S1). The differences in the levels of the three unsaturated FAs among the three tested environments were not statistically significant, as indicated by the coefficient of variation (Additional file 1: Table S1), suggesting that the contents of the three unsaturated FAs coincided with the three study environments. Among the three tested unsaturated FAs, LNA was the least variable, whereas OA and LLA had more variation (Additional file 1: Table S1). The wide range of variations in content of the three unsaturated FAs in the 194 accessions was found and continuous the distribution of the three unsaturated FAs in this GWAS panel was generally normal.

### Distribution of SNP markers and LD

The genotyped samples consisted of a set of 194 soybean germplasms representing a diverse range of unsaturated FA contents (Additional file 2: Supplementary Data). A total of 36,981 SNPs with minor allele frequencies (MAF) > 0.04 and missing data of < 10% were used for the estimation of LD of the soybean accessions. These SNP markers were evenly distributed among 20 soybean chromosomes, resulting in an average SNP density of 1 SNP every 25.67 kb in the unique genomic region. The mean LD was 227 kb (Additional file 3: Figure S1).

### QTNs associated with the content of three unsaturated FAs identified by GWAS

**Fig. 2:**
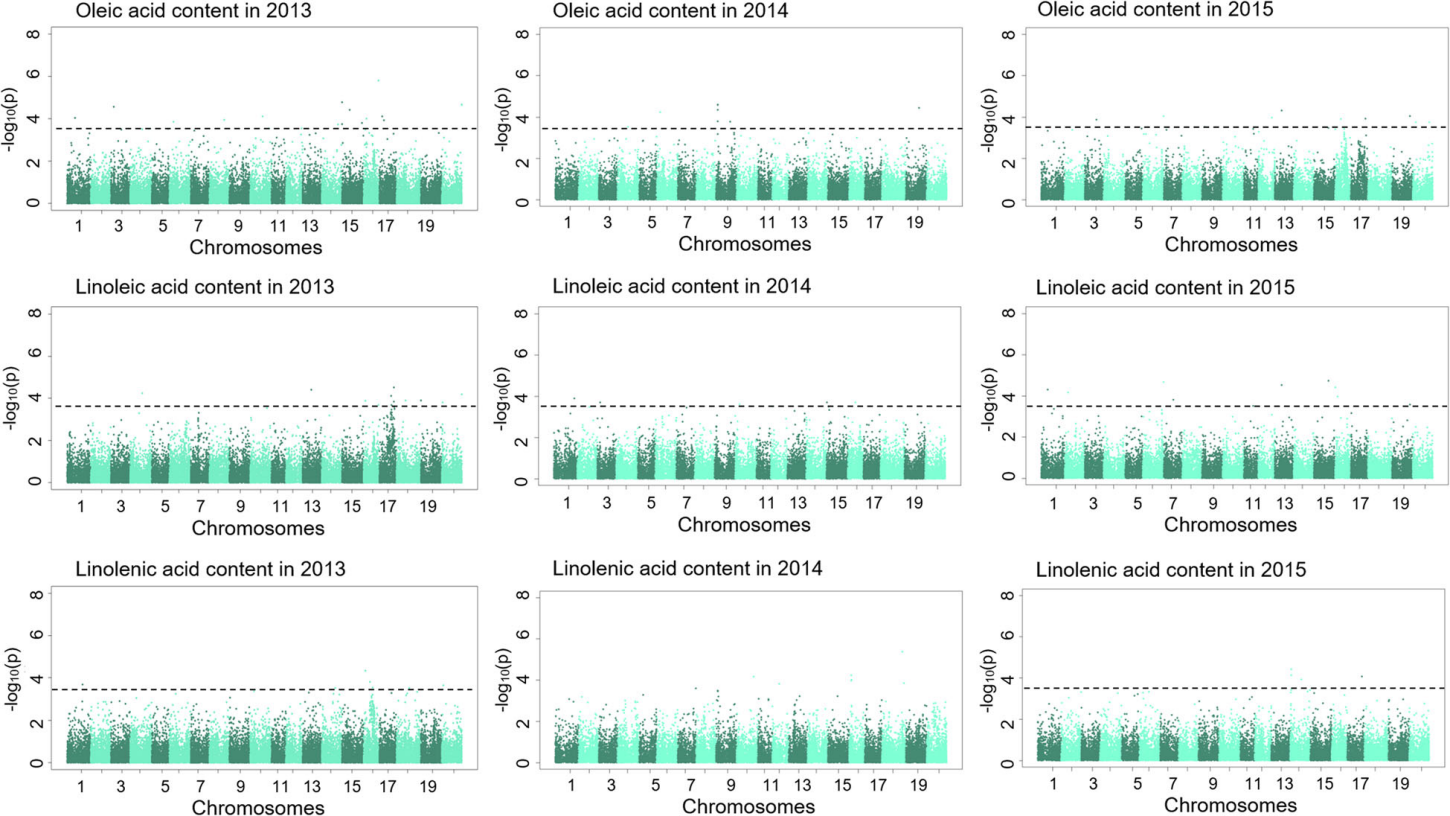
Manhattan plot of association mapping for unsaturated FA content in soybean seed

A total of 120 SNPs that covered all 20 soybean chromosomes and represented 62 genomic regions were determined to be associated with the contents of three kinds of unsaturated FAs in soybean seeds by GWAS (Table 1, Fig. 2). For OA content in soybean seed, 15, 11, and 29 SNPs were detected in 2013, 2014, and 2015, respectively, which represented 37 genomic regions covering 18 soybean chromosomes. For LLA content, 10, 11, and 29 SNPs were detected in 2013, 2014, and 2015, respectively, which represented 42 genomic regions covering 19 soybean chromosomes. For LNA content, 4, 35, and 7 SNPs were detected in 2013, 2014, and 2015, respectively, which represented 16 genomic regions covering 14 soybean chromosomes.

**Table 1:** Peak SNP, beneficial allele and candidate genes associated with unsaturated FA identified by GWAS

Trait	Year	Chr.	Peak SNP	Peak SNP position	Number of significant SNPs	-log (*P*-value) of Peak SNP	R2 (%) of Peak SNP	Allele 1	Allele 2	Average trait value of accessions with allele 1	Average trait value of accessions with allele 2	Average value of population	IDs of FA related genes	Known QTL	Reference
Oleic acid	2013	1	rs18996061	18,996,061	1	4.05	16.08	T	A	20.28	31.46	20.42			
	2013	3	rs6779592	6,779,592	1	4.58	17.65	A	C	20.31	29.22	20.42			
	2013	6	rs10058820	10,058,820	1	3.87	15.53	G	C	20.25	25.68	20.42			
	2013	8	rs35562739	35,562,739	1	3.96	15.81	A	T	20.26	28.49	20.42			
	2013	10	rs30082069	30,082,069	1	4.12	16.27	C	T	20.23	23.24	20.42			
	2013	14	rs39135305	39,135,305	1	3.73	15.11	C	T	20.26	26.03	20.42			
	2013	15	rs259146	259,146	2	4.77	18.25	A	T	20.23	23.61	20.42			
	2013	15	rs18512918	18,512,918	1	4.43	17.19	T	C	20.20	24.44	20.42			
	2013	15	rs47976104	47,976,104	1	3.80	15.34	G	T	20.21	23.96	20.42			
	2013	16	rs6724381	6,724,381	1	4.01	15.95	T	C	20.33	35.33	20.42			
	2013	16	rs36081420	36,081,420	1	5.80	21.44	T	G	18.79	20.77	20.42	Glyma.16G200200Glyma.16G003500		
	2013	17	rs7339569	7,339,569	1	4.12	16.27	C	A	20.26	27.85	20.42			
	2013	17	rs10758737	10,758,737	1	3.93	15.71	G	T	20.25	27.28	20.42			
	2013	20	rs47531251	47,531,251	1	4.67	17.92	G	A	20.26	28.44	20.42	Glyma.20G158300Glyma.20G245500		
	2014	3	rs35644650	35,644,650	2	5.01	23.46	G	T	21.21	25.89	21.42			
	2014	6	rs8371282	8,371,282	1	4.24	21.62	G	T	21.31	24.07	21.42			
	2014	7	rs18395919	18,395,919	1	4.13	21.38	G	T	21.27	24.73	21.42	Glyma.07G151300		
	2014	8	rs20308530	20,308,530	3	5.14	23.76	A	C	21.20	25.89	21.42	Glyma.08G233200		
	2014	9	rs34018579	34,018,579	5	3.77	20.56	C	A	21.19	24.22	21.42			
	2014	15	rs33757180	33,757,180	1	3.68	20.34	C	A	21.28	24.35	21.42			
	2014	16	rs33751855	33,751,855	1	3.63	20.24	A	G	21.36	26.92	21.42			
	2014	18	rs5683686	5,683,686	1	3.60	20.16	G	T	21.28	27.54	21.42	Glyma.18G062000	Ole 3–2	
	2014	19	rs31881460	31,881,460	1	4.45	22.11	G	T	21.16	23.78	21.42		Ole 2–2	
	2015	1	rs50012108	50,012,108	1	4.39	11.38	T	A	19.05	22.51	19.19			
	2015	3	rs35817332	35,817,332	2	3.76	9.71	G	A	18.95	22.22	19.19			
	2015	4	rs29355727	29,355,727	1	5.66	14.79	C	T	18.95	27.66	19.19			
	2015	6	rs50110801	50,110,801	2	4.05	10.45	T	A	18.96	21.08	19.19			
	2015	7	rs3937684	3,937,684	1	3.64	9.40	G	T	19.07	25.48	19.19	Glyma.07G046200	Ole 7–1	
	2015	11	rs24645193	24,645,193	1	4.16	10.75	G	T	19.07	21.62	19.19			
	2015	12	rs32443263	32,443,263	1	3.96	10.23	G	T	18.94	22.04	19.19			
	2015	13	rs16802809	16,802,809	2	4.32	11.17	T	C	18.88	21.57	19.19	Glyma.13G068600Glyma.13G069100		
	2015	15	rs1748955	1,748,955	1	4.58	11.86	C	A	19.01	26.82	19.19		Ole 1–4	
	2015	16	rs36081420	36,081,420	2	6.29	16.53	T	C	19.10	34.81	19.19	Glyma.16G200200Glyma.16G003500		
	2015	17	rs35024325	35,024,325	2	3.92	10.12	C	A	18.93	22.96	19.19			
	2015	18	rs12585602	12,585,602	1	4.09	10.56	T	A	19.04	28.45	19.19			
	2015	19	rs43329219	43,329,219	1	4.04	10.46	G	A	18.90	22.40	19.19		Ole 2–2	
	2015	20	rs47531251	47,531,251	4	4.84	12.56	G	A	18.96	27.37	19.19	Glyma.20G158300Glyma.20G245500		
Linoleic acid	2013	4	rs29355727	29,355,727	1	4.24	20.13	T	C	46.08	55.59	55.34			Reinprecht et al. 2006
	2013	13	rs20383965	20,383,965	1	4.40	20.61	T	G	48.18	55.57	55.34			Hyten et al. 2004
	2013	16	rs4446239	4,446,239	1	3.88	19.13	T	G	52.43	55.64	55.34			
	2013	17	rs28191708	28,191,708	1	4.12	19.79	C	T	54.12	55.81	55.34			
	2013	17	rs28979581	28,979,581	1	3.68	18.55	G	T	54.18	55.80	55.34			
	2013	17	rs34633822	34,633,822	1	4.52	20.96	C	T	52.36	55.70	55.34			
	2013	17	rs35259287	35,259,287	1	3.85	19.02	T	C	51.76	55.53	55.34			Kim et al. 2010
	2013	18	rs21573804	21,573,804	1	3.90	19.18	A	T	52.35	55.58	55.34			
	2013	19	rs577646	577,646	1	3.90	19.18	A	G	54.00	55.63	55.34			
	2013	20	rs1608443	1,608,443	1	3.81	18.90	G	C	54.76	57.56	55.34			
	2013	16	rs36081420	36,081,420	1	3.67	18.52	C	T	43.10	55.41	55.34	Glyma.16G200200Glyma.16G003500		Diers and Shoemaker 1992
	2013	20	rs47531251	47,531,251	1	4.19	20.01	A	G	50.96	55.51	55.34	Glyma.20G158300Glyma.20G245500		
	2014	1	rs49504957	49,504,957	1	3.90	19.01	G	T	54.09	55.35	54.26			
	2014	2	rs4953186	4,953,186	1	4.23	18.40	G	T	54.11	55.78	54.26			
	2014	3	rs6481810	6,481,810	1	4.11	18.10	C	T	53.16	54.43	54.26			Hyten et al. 2004
	2014	8	rs39589269	39,589,269	3	3.82	17.42	G	T	54.15	55.47	54.26			
	2014	9	rs20595231	20,595,231	1	3.70	17.14	T	C	51.27	54.31	54.26			
	2014	10	rs10278177	10,278,177	1	3.70	17.14	A	T	51.93	54.41	54.26			
	2014	13	rs31318746	31,318,746	1	3.70	17.13	T	C	50.54	54.40	54.26		Linole 2–1	
	2014	15	rs1064298	1,064,298	1	3.65	17.02	C	T	54.07	55.64	54.26			
	2014	16	rs18741800	18,741,800	1	3.60	16.90	A	C	52.28	54.37	54.26			
	2015	1	rs50012108	50,012,108	3	6.40	17.63	A	T	53.42	56.38	56.26			
	2015	2	rs7975863	7,975,863	1	5.16	14.25	T	C	53.36	56.44	56.26			Hyten et al. 2004
	2015	3	rs35817332	35,817,332	1	4.80	13.29	A	G	53.24	56.48	56.26			
	2015	4	rs29355727	29,355,727	1	4.73	13.11	T	C	49.13	56.93	56.26			
	2015	5	rs26200085	26,200,085	2	4.67	12.70	C	T	52.23	56.33	56.26			
	2015	6	rs50110801	50,110,801	3	4.52	12.56	A	T	50.03	56.46	56.26			
	2015	7	rs22961030	22,961,030	2	4.41	12.28	T	C	53.64	56.41	56.26			
	2015	8	rs35562739	35,562,739	1	4.26	11.87	T	A	49.76	56.37	56.26			
	2015	9	rs30794068	30,794,068	1	4.26	11.86	T	G	53.05	56.36	56.26			
	2015	11	rs6577617	6,577,617	1	4.24	11.82	T	G	52.42	56.39	56.26			
	2015	13	rs16802831	16,802,831	2	4.20	11.72	T	C	54.59	56.48	56.26		Linole 6–9	
	2015	14	rs39135305	39,135,305	1	4.16	11.62	T	C	52.44	56.41	56.26		Linole 2–1	
	2015	15	rs35324780	35,324,780	2	3.97	11.12	T	C	52.96	56.43	56.26			
	2015	16	rs36081420	36,081,420	4	3.83	10.43	C	T	44.07	56.33	56.26	Glyma.16G200200Glyma.16G003500		
	2015	17	rs34339550	34,339,550	1	3.68	10.40	G	A	49.88	56.33	56.26			Bachlava et al. 2009
	2015	18	rs39638216	39,638,216	1	3.67	10.37	T	G	52.95	56.38	56.26			Hyten et al. 2004
	2015	19	rs14807896	14,807,896	1	3.59	10.16	T	T	53.69	56.36	56.26			
	2015	20	rs47531251	47,531,251	1	3.58	10.14	A	G	50.47	56.36	56.26	Glyma.20G158300Glyma.20G245500		
Linolenic acid	2013	1	rs37146219	37,146,219	1	3.68	10.98	T	C	6.69	8.82	8.73			
	2013	16	rs3989930	3,989,930	1	4.33	13.01	G	T	8.59	10.37	8.73			
	2013	16	rs15449450	15,449,450	1	3.81	11.39	A	G	8.23	9.05	8.73			
	2013	20	rs3008708	3,008,708	1	3.63	10.85	G	A	5.01	8.83	8.73			
	2014	2	rs35057707	35,057,707	23	3.66	30.58	G	A	8.09	10.52	8.13			
	2014	4	rs48837377	48,837,377	1	3.77	30.79	T	C	7.26	8.21	8.13			
	2014	7	rs43158730	43,158,730	1	3.60	30.44	C	T	8.10	10.40	8.13	Glyma.07G254500		
	2014	10	rs41662636	41,662,636	1	4.16	31.58	G	T	8.00	10.04	8.13			
	2014	12	rs16701034	16,701,034	1	3.81	30.88	G	A	8.05	9.13	8.13			
	2014	16	rs5443771	5,443,771	3	4.01	31.27	G	T	8.02	8.27	8.13			
	2014	18	rs52833743	52,833,743	4	3.85	30.94	A	C	8.02	8.79	8.13		Linolen 4–2	Panthee et al. 2006
	2014	19	rs21750320	21,750,320	1	3.83	30.90	G	C	8.05	9.66	8.13			
	2015	13	rs14900419	14,900,419	1	3.98	10.27	A	T	9.10	10.93	9.19			
	2015	14	rs45949815	45,949,815	4	3.81	9.83	T	A	9.11	11.59	9.19	Glyma.14G194300		
	2015	17	rs35024325	35,024,325	1	4.06	10.48	C	T	9.12	10.62	9.19			
	2015	19	rs13718585	13,718,585	1	3.94	10.17	A	C	7.97	9.27	9.19

To confirm the beneficial alleles of each SNP associated with unsaturated FA content in soybean seeds, the average FA content of OA, LLA, and LNA of soybean accessions that carried each allele of peak SNPs were analyzed. Of the 73 peak SNPs that were associated with the three target traits, 73 beneficial alleles were identified. The ‘Allele 1’ of each peak SNP in Table 1 showed the effect of increasing the phenotypic value of unsaturated FA content. Inversely, the ‘Allele 2’ of each peak SNP in Table 1 was showed the effect of decreasing the phenotypic value of unsaturated FA content (Table 1). Concretely, for seed OA content, the average phenotypic value of accessions with ‘Allele 1’ were 1.98 to 15.71% higher than that with ‘allele 2’ (Table 1). For seed LLA content, the average phenotypic value of accessions with ‘Allele 1’ were 1.26 to 12.31% higher than that of accessions with ‘Allele 2’ (Table 1). For seed LNA content, the average phenotypic values of accessions with ‘Allele 1’ were 0.25 to 3.82% higher than that with ‘Allele 2’ (Table 1). To increase of unsaturated FA concentrations in soybean oil is desirable to improve human cardiovascular health. However, LNA increases the oxidation of food oils, causing an off-flavor and reducing the shelf life of the oil [3]. Therefore, the important focus of soybean breeding for FAs is to increase the OA and LLA acid contents and to reduce LNA content in seed oil. Hence, the ‘Allele 1’ of each peak SNP associated with OA and LLA content were beneficial alleles and the ‘Allele 2’ of each peak SNP associated with LNA content were beneficial alleles for marker-assistant selection (MAS) for the improvement of the seed FA compositions in soybean oil (Table 1).

**Fig. 3:**
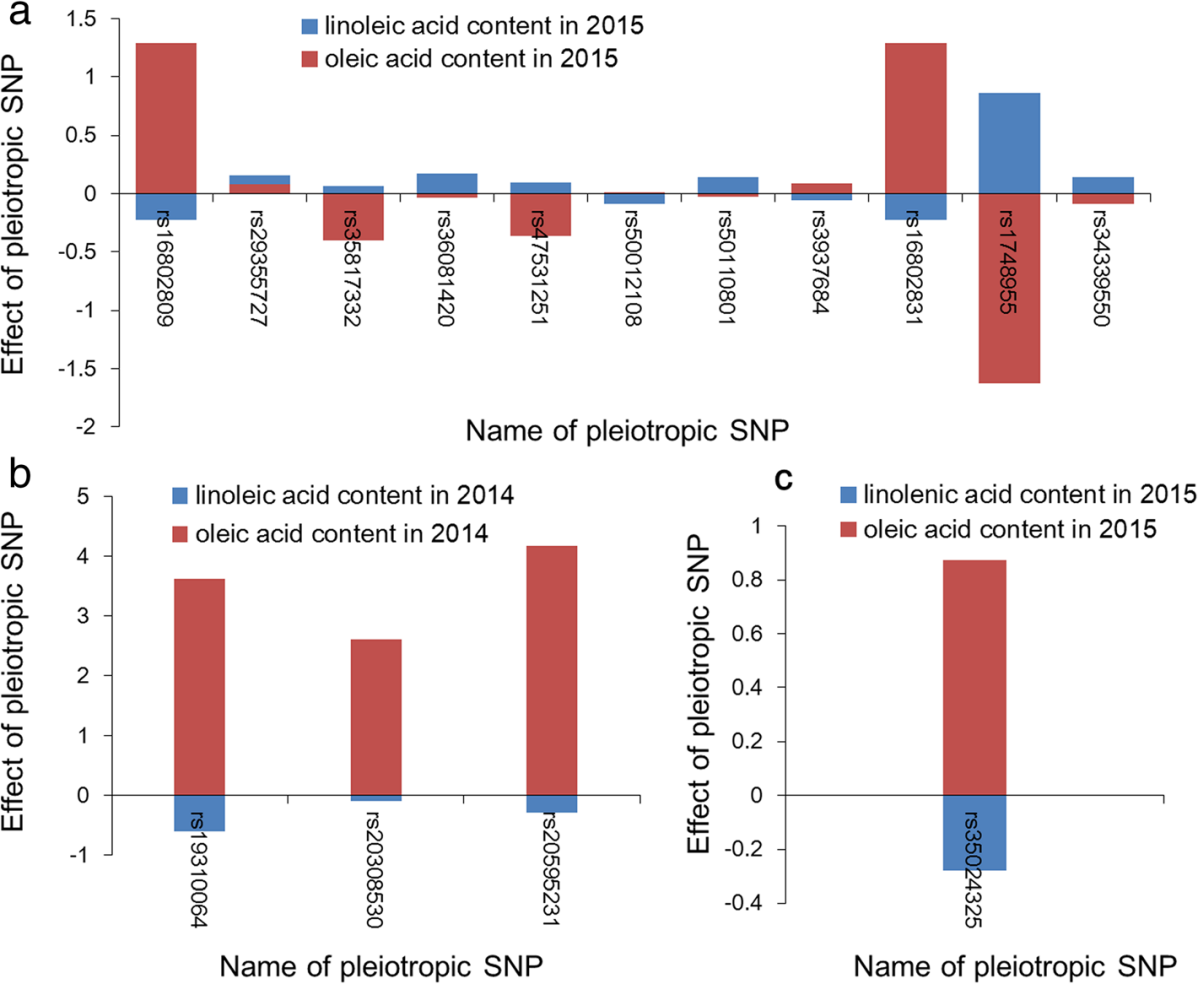
The allelic effects of the pleiotropic SNPs. **a**: Allelic effects of pleiotropic SNPs underlying oleic- and linoleic- acid content in 2014. **b**: Allelic effects of pleiotropic SNPs underlying oleic- and linoleic- acid content in 2014. **c**: Allelic effects of pleiotropic SNPs underlying oleic- and linolenic- acid content in 2015

Fifteen pairs of pleiotropic loci were detected both in 2014 and 2015. Fourteen pairs of pleiotropic loci (showing peak SNP here rs50012108 on Chr.01, rs35817332 on Chr.03, rs29355727 on Chr.04, rs50110801 on Chr.06, rs16802809 on Chr.13, rs36081420 on Chr.16 and rs47531251 on Chr.20) were found controlling seed OA and LLA content and one pair of pleiotropic loci (rs35024325 on Chr.17) was found controlling OA and LNA content, simultaneously. The allelic effects of all the pleiotropic SNP were analyzed (Fig. 3). Eleven SNPs were detected controlling OA and LLA content in soybean seed in 2015. Except for rs29355727 on Chr. 04, the direction of the contribution to the phenotype value of all the rest 10 SNPs was opposite according to the value of each allelic effect. The same tendency was also found in 2014. All the four SNPs identified in 2014 that associated with OA and LLA content of soybean seed showed the opposite direction of the contribution to the phenotype value. There into, the allelic effect values of SNP loci, rs16802809 on Chr.13, rs50012108 on Chr.01, rs3937684 on Chr.07, and rs16802831 on Chr.13 were positive for OA content and negative for LLA content in 2015. The allelic effect values of three SNP loci, rs19310064 on Chr.08, rs20308530 on Chr.08 and rs20595231 on Chr.09, were positive for OA content and negative for LLA content in 2014. Therefore, the above seven SNPs could be useful for assisted selection for soybeans with high OA content and low LLA content at the same time.

### Predicting and verifying of candidate genes for unsaturated FAs

A total of 1283 genes were found in the 100-kb flanking genomic region of each peak SNP related to three unsaturated FAs according to soybean genome v2.1 (Phytozome 10.0) (Additional file 4: Table S2). Of them, 915 genes were categorized into 29 groups based on BLAST sequence homologies and gene ontology (GO) annotations using MapMan [21] (Additional file 5: Figure S2). Thirty-four genes involved in lipid metabolism. Among the 34 lipid metabolism-related genes, 12 genes that were involved in unsaturated FA synthesis of soybean were considered as candidates for expression profile analysis during the accumulation of soybean seeds (Table 1).

**Fig. 4:**
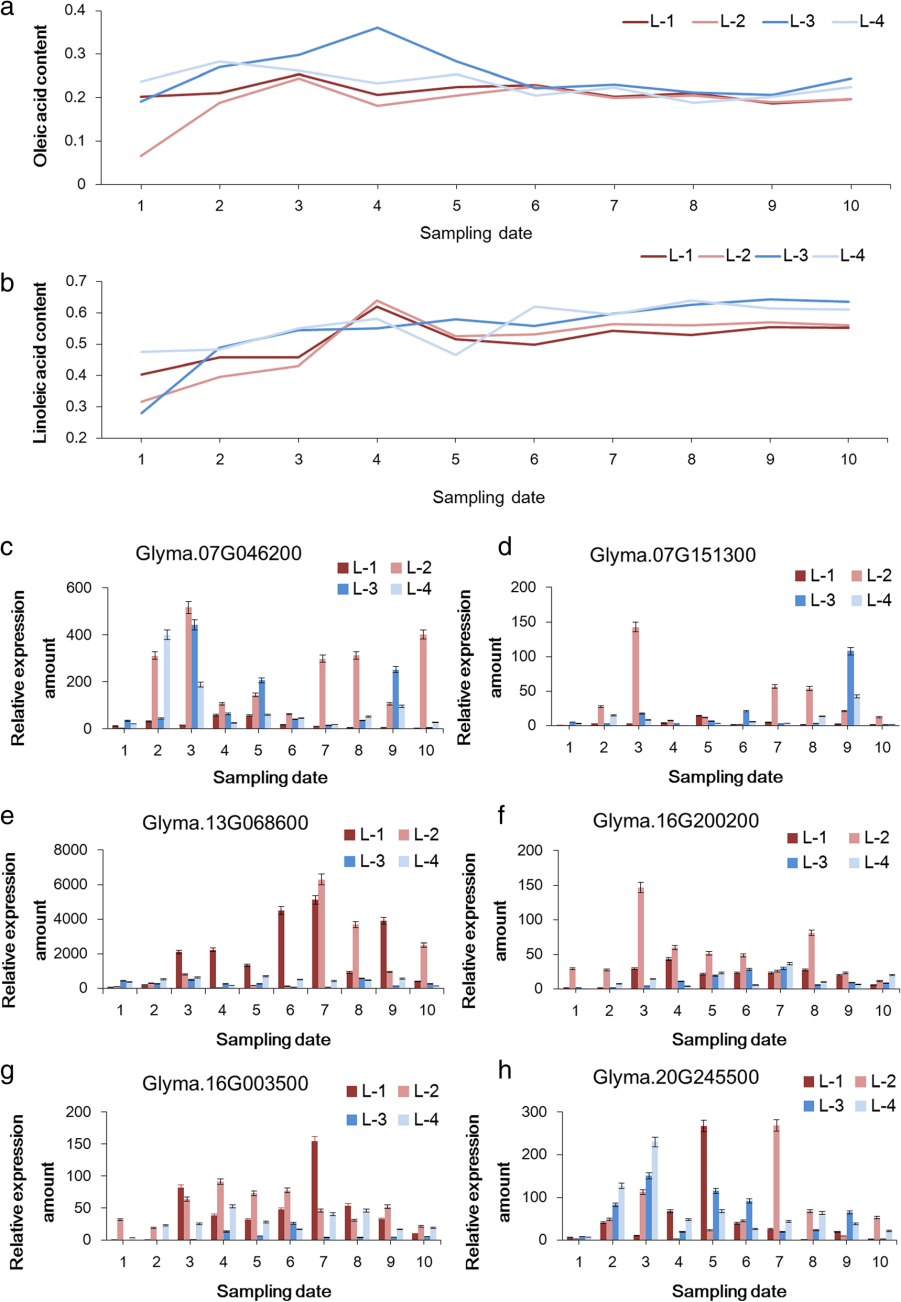
The relationship between expression level of candidate genes and oleic acid content as well as linoleic acid content detected during the seed developmental stages in characteristic soybean accessions. **a**-**b**: the dynamic oleic- and linoleic- acid content of four characteristic soybean accessions. For the ten sampling points on x axis, 1–4 corresponds with R5 stage, 5–6 corresponds with R6 stage, 7–8 corresponds with R7 stage, 9–10 corresponds with R8 stage. **c**-**h**: The relative expression patterns of candidate genes that are possibly responsible for oleic- and/or linoleic- acid content

**Fig. 5:**
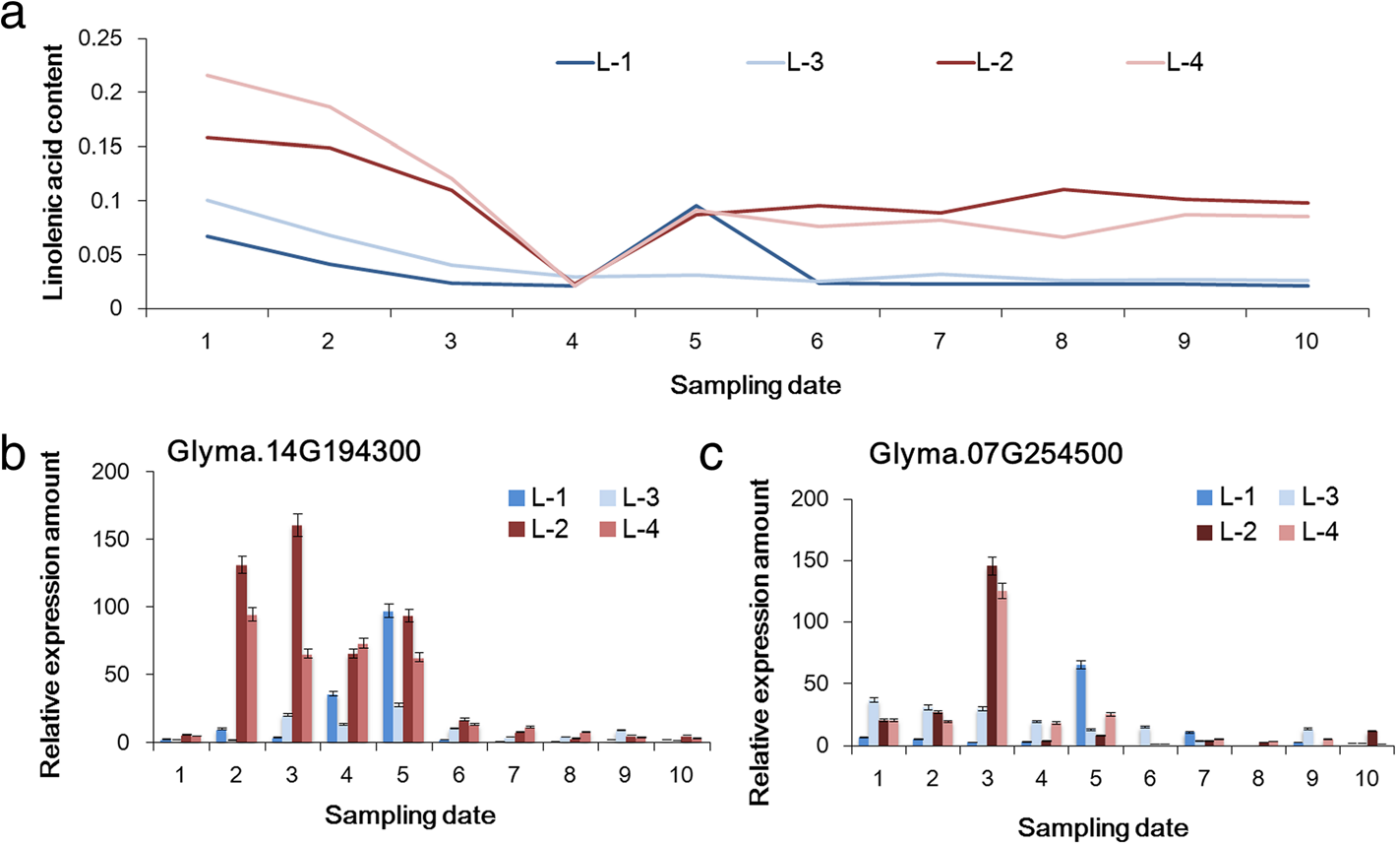
The relationship between expression level of candidate genes and linolenic acid content detected during the seed developmental stages in characteristic soybean accessions. **a**: the dynamic linolenic acid content of four characteristic soybean accessions. **b**-**c**: The relative expression patterns of candidate genes that are possibly responsible for linolenic acid content

To predict possible roles of candidate genes on unsaturated FA content regulation, dynamic accumulation of three kinds of FAs in four characteristic soybean accessions were determined during the development stages of seeds (R5-R8) (Figures 4a-b and 5a). Furthermore, a quantitative RT-PCR was performed to analyze the expression of eight candidate genes in the same four soybean accessions from R5 to R8. All the candidates could express in immature and mature soybean seeds, although the expression levels were largely different among soybean accessions and development stages (Figs. 4c-h and 5b-c).

According to the result of quantitative RT-PCR, the expression levels of *Glyma.13G068600* and *Glyma.16G003500* in low OA carriers (‘Line3’ and ‘Line 4’) of each sampling stage were much higher than those in high OA carriers (‘Line1’ and ‘Line2’). The expression levels of *Glyma.16G200200* in low OA carriers (Lines 3 and 4) were much higher than those in high OA carriers (‘Line1’ and ‘Line2’) during R5 and R6 stages. The expression levels of *Glyma.07G151300* in high OA carriers (‘Line1’ and ‘Line2’) were much higher than those in low OA carriers (‘Line3’ and ‘Line4’) during R7 stage. The similar tendency was found in LLA for the genes mentioned above. No obvious rules were found in the dynamic expression of the other candidate genes.

For LNA, the peak expression of *Glyma.14G194300* and *Glyma.07G254500* occurred at the early developmental stages of soybean seeds (R5 to R6 stages), which is similar to the trend of dynamic changes in the phenotype of LNA. The expression levels of *Glyma.14G194300* in high LNA carriers (‘Line1’ and ‘Line3’) were much higher than those in low LNA carriers (‘Line2’ and ‘Line4’) indicating that the gene might promote the accumulation of LNA. The obvious difference in expression of *Glyma.07G254500* between high and low LNA carriers was detected only in R5 stage. The regulation pattern of candidate genes could not be sufficient to determine only by analyzing the expression patterns. Therefore, correlation analysis was conducted between on the four tested strains of dynamic phenotypic data of unsaturated FA content and the relative expression of candidate genes (Table 2).

**Table 2:** Correlation coefficient (R) between phenotypic value and relative expression amount of candidate genes during the development of soybean seed in the representative accessions with relative higher and lower oleic- and linolenic- acid content

Relative expression amount	Sample no.	Oleic acid content				Relative expression amount	Oleic/Linoleic acid content				Relative expression amount	Linolenic acid content			
		L-1	L-2	L-3	L-4		L-1a	L-2	L-3	L-4		L-1	L-2	L-3	L-4
Glyma.07G 046200	L-1	0.24				Glyma.16G200200	0.32/0.63*				Glyma.07G254500	−0.33			
	L-2		0.57*					0.46/−0.09					0.05		
	L-3			0.27					−0.06/0.36					−0.33	
	L-4				0.68**					0.05/0.07					0.10
Glyma.07G 151,300	L-1	0.21				Glyma.16G003500	0.22/0.23				Glyma.14G194300	0.84***			
	L-2		0.49					0.33/0.44					0.21		
	L-3			−0.31					0.1/0.23					0.78***	
	L-4				−0.27					−0.17/0.36					0.20
Glyma.13G 068600	L-1	0.01				Glyma.20G245500	0.24/0.12								
	L-2		0.18					0.33/0.12							
	L-3			0.05					0.28/0.10						
	L-4				0.21					0.59*/−0.23					

Note: R **=** 0.54936 (df = 8, α = 0.1); R **=** 0.63190 (df = 8, α = 0.05); R **=** 0.76459 (df = 8, α = 0.01)

According to correlation analysis results, for the dynamic content of OA in soybean seeds, the positive correlations were found between the relative expression amount of *Glyma.07G046200* and *Glyma.20G245500* and dynamic OA content during the development of seeds of four tested soybean accessions. There into, the relationship between the relative expression amount of *Glyma.07G046200* and dynamic OA content of ‘Line1’ and ‘Line3’ was significantly positive correlation. Meanwhile, the relationship between the relative expression amount of Glyma.20G245500 and dynamic OA content of ‘Line4’ was significant positive correlation indicating that *Glyma.07G046200* and *Glyma.20G245500* genes should promote the OA accumulation in soybean seed. Positive correlations were found between the relative expression amount of *Glyma.13G068600* and *Glyma.16G200200* and dynamic OA content of ‘Line1’ and ‘Line2’. No obvious correlation was found between the relative expression amount of *Glyma.13G068600* and *Glyma.16G200200* and dynamic OA content of ‘Line3’ and ‘Line4’, indicating that these two genes might have the effect of increasing the OA accumulation in high OA carrier germplasms and the effect was not obvious in low OA carrier germplasms. The dynamic OA content of high OA carrier germplasms (‘Line1’ and ‘Line2’) showed positive correlation with the relative expression amount of *Glyma.07G151300*. On the contrary, the dynamic OA content of low OA carrier germplasms (‘Line3’ and ‘Line4’) showed negative correlation with that of *Glyma.07G151300*. The result suggested that *Glyma.07G151300* could promote the accumulation of OA in germplasms with relatively higher OA content and suppress that in germplasms with relatively lower OA content. The relative expression amount of *Glyma.13G068600* showed a weak positive correlation with the dynamic OA content of all the four tested accessions suggesting that the gene has less influence on the OA accumulation.

For the dynamic LLA content, the relative expression amount of *Glyma.20G245500* was positively correlated with the dynamic LLA content of four tested accessions. Among the four positive relationships, a significantly positive correlation was found between the relative expression amount and LLA content of ‘Line4’. It is speculated that *Glyma.20G245500* might have a positive regulatory effect on LLA accumulation. The relative expression amount of *Glyma.16G003500* showed a positive correlation relationship with LLA content of Lines 1 and 2 (high LLA carrier) but showed a weak positive or negative correlation relationship with ‘Line3’ and ‘Line4’ (low LLA carrier) indicating that *Glyma.16G003500* has the effect of increasing the LLA accumulation in relatively higher LLA germplasms with and has no effect in relatively lower LLA germplasms. Positive correlations were found between the relative expression amount of *Glyma.16G200200* and dynamic LLA content of ‘Line1’ and ‘Line3’. There into, the very significant positive correlation was found between the relative expression amount of *Glyma.16G200200* and dynamic LLA content of ‘Line1’. Meanwhile, no relation was found between the relative expression amount of *Glyma.16G200200* and dynamic LLA content of ‘Line2’ and ‘Line4’.

For dynamic content of LNA, the dynamic LNA content of germplasms with lower LNA content (‘Line1’ and ‘Line3’) showed negative correlation with the relative expression amount of *Glyma.07G254500*. On the contrary, no obvious correlation was found between the relative expression amount of *Glyma.07G254500* and the dynamic LNA content of high LNA carrier germplasms (‘Line2’ and ‘Line4’). The result suggested that *Glyma.07G254500* could suppress the LNA accumulation in germplasms with lower OA content. The relative expression amount of *Glyma.14G194300* was very significantly correlated with the dynamic LNA content of low LNA carrier germplasms (‘Line1’ and ‘Line3’). While in germplasms with higher LNA content (‘Line2’ and ‘Line4’), the positive correlation was not obvious. The result suggested that *Glyma.14G194300* might be involved in the accumulation of LNA content in germplasms with lower phenotypic value. But the regulation effect was not obvious in high LNA content germplasms.

## Discussion

Different concentration and relative proportions of unsaturated FA in soybean seed may play a decisive role in the nutritional value of soybean oil. Breeding soybean lines with improved unsaturated fatty acid level was primary goal of some soybean breeding programs. In the present study, a total of 194 samples, mainly collected inside China, were evaluated unsaturated fatty acid level. Among these, three and one accessions were found with higher OA (> 30%) and lower LNA level (< 4%), respectively, which both have perfect agronomic traits. Therefore, these accessions with reasonable unsaturated FA level have great potential value for in the future MAS.

To date, about 200 unsaturated FA QTL have been reported (www.soybase.org) based on linkage analysis, most of which were identified via different cross populations constructed by some specific materials. Of these identified QTLs, many were related to the mutant of two major delta 12 FA desaturase enzymes genes (*FAD2–1A* and *FAD2–1B*), which participated in conversion of OA precursors to LLA precursors in the soybean seed lipid biosynthesis pathway [22]. In this study, 11 and 29 SNPs located 23 genomic regions of 16 chromosomes, 11 and 29 SNPs located 27 genomic regions of 19 chromosomes, and 35 and 7 SNPs located 12 genomic regions of 12 chromosomes, were identified to be associated with OA, LLA, and LNA level in 2014 and 2015, respectively. Most of these genomic regions were firstly reported to be associated with unsaturated FA concentrations. Among the 11 and 29 association signals for OA content in 2014 and 2015, a total of five SNPs were overlapped with or near the known OA QTL (Table 1). For instance, two genomic regions in rs31881460 and rs43329219 of Chr.08 were significantly associated with OA level, and the association between these two genomic regions and OA content had been verified by previous studies [23]. Similarly, three SNPs (rs3937684, rs1748955, and rs5683686 located on Chr7, Chr15, and Chr18, respectively) of this study were located near the two marker intervals reported by other studies [24]. For the identified 11 and 29 association signals for LLA in 2014 and 2015, one and two genomic regions defined by SNP marker rs31318746, and rs16802831and rs39135305, have similar genomic regions with that of the previous study [25]. Panthee et al. (2006) [26] reported one LNA QTL Linolen4–2, has similar genomic regions as that of this study (rs52833743). These consistent genomic regions across China and North American genetic background might play important roles in regulating unsaturated FA level.

*FAD2–1A* and *FAD2–1B* were expressed in developing seeds and many studies have shown that *FAD2–1A* and *FAD2–1B* could take part in regulating unsaturated FA concentration [27]. Therefore, most soybean breeder selected *FAD2–1A* and *FAD2–1B* as target genes to improve unsaturated FA level, especially OA level. Except for these two significant genes, other genes were still difficult to predict and confirm as the candidates. However, GWAS was an effective way to identify and confirm candidate genes especially within 150–200 kb LD block [19]. In this study, the mean LD was 227 kb, which ensure the candidate gene to be effectively identified. Therefore, we screened candidate genes in the 100-kb flanking regions up- and downstream of OA, LLA, and LNA peak SNPs. We first focused whether the *FAD* family genes existed in LD blocks when we predicted candidate genes. There were 20 *FAD* genes in soybean genome including *FAD2*, *FAD4*, *FAD6* and *FAD8* (Phytozome 10.0). However, most of the *FAD* genes were beyond or just barely beyond the LD interval except for two *FAD8* genes (*Glyma.07G151300* and *Glyma.14G194300*). Our study found that the biological function of different copies of *FAD8* genes in soybean was differentiated. Based on our results, *Glyma.07G151300* promotes OA accumulation in higher OA germplasms and suppresses that in lower OA germplasms. *Glyma.14G194300* might be involved in the accumulation of LNA content in germplasms with lower phenotypic value, but the regulation effect was not obvious in high LNA content germplasms. The *FAD8* gene was identified as a light- and temperature-sensitive ω3 desaturase and the activity of which was cold inducible [28–30]. The functional differentiation *FAD8* gene among different germplasms might be due to differential responses to light and temperature conditions. Previous studies suggest that ubiquitination may play an important role in plant tolerance against various abiotic stresses [31–34]. Also the ubiquitin-dependent protein degradation pathway is involved in photomorphogenesis, hormone regulation, floral homeosis, senescence, and pathogen defense [35, 36]. Interestingly, we found a ubiquitin-conjugating enzyme gene (*Glyma.07G046200*) and a putative proteasome (*Glyma.20G245500*) were promote the OA accumulation in soybean seed meantime. It was the first time to find the function of ubiquitination on the accumulation of FA in soybean. However, the accurate function and the regulatory network of the candidates will be discussed in a future work.

## Conclusions

A total of 149 SNPs that represented 73 genomic regions were found to be associated with the unsaturated FA contents in soybean seeds in 2013, 2014 and 2015 according to the results of GWAS. Twelve novel genes were predicted to be involved in unsaturated FA synthesis in soybean. Of them, eight genes might be involved in unsaturated FAs regulation simultaneously but work in very different ways revealed by the relationship between expression pattern of the candidate genes and the accumulation of unsaturated FAs. The beneficial alleles and candidate genes identified might be valuable for improving marker-assisted breeding efficiency and exploring the molecular mechanisms underlying unsaturated fatty acid of soybean.

## Methods

### Plant materials, field design, and phenotypic evaluation

A total of 194 Chinese representative accessions, comprising landraces and elite cultivars, were collected from more than 15,000 samples at the Chinese National Soybean GeneBank (CNSGB), were planted at Harbin in 2013, 2014, and 2015. Field trials were conducted using single-row plots (3-m long and 0.65-m between rows) and a randomized complete block design with three replicates per tested environment.

For association mapping, 10 randomly selected plants from each line in each plot were collected to evaluate the levels of unsaturated FAs after full maturity. To study the expression patterns of candidate genes, four characteristic accessions with relatively higher and lower LNA, OA, and LLA contents within the association panel, including ‘Line1’ (2.10, 19.63, and 55.34%), ‘Line2’ (9.79, 19.71, and 56.09%), ‘Line3’ (2.63, 24.38, and 63.65%), and ‘Line4’ (8.52, 22.51, and 61.06%) were grown in pots in 2016. Pods were picked from the fifth to the seventh nodes of the main stem every seven days from the R5 to the R8 stages of plant, with three replicates for each sample. Seeds in pods were used for the isolation of total RNA and determination of unsaturated FA contents. The unsaturated FA contents in tested samples were determined using gas chromatography (GC-14C, Shimadzu Company, Japan) according to our previous method [37].

### SNP genotyping data collection

Genomic DNA was extracted from fresh leaves of each experimental plant using the CTAB method [38], and then were sequenced by specific locus amplified fragment sequencing (SLAF-seq) methodology [39]. According to the preliminary evaluation of soybean reference genome, two restriction enzymes, *Mse* I (EC 3.1.21.4) and *Hae*III (EC: 3.1.21.4) (Thermo Fisher Scientific, Inc., Waltham, MA, USA), were utilized to generate 50,000 sequencing tags (about 300–500 bp in length) from all tested samples. The sequencing libraries of each accession were constructed based on the sequencing tags, which were distributed in unique genomic regions of 20 soybean chromosomes. For each accession library, the 45-bp sequence reads at both ends of the sequencing tags were obtained through a barcode method using an Illumina Genome Analyzer II system (Illumina Inc., San Diego, CA, USA). The Short Oligonucleotide Alignment Program 2 (SOAP2) software was used to align raw paired-end reads onto the soybean reference genome [40]. The SLAF groups were defined using the raw reads in the same genomic position. More than 58,000 high-quality SLAF tags were obtained from each tested sample. In SNP calling, a minor allele frequency (MAF) threshold was set at 0.04. If depth of a minor allele was larger than 1/3 of sample total depth, the genotype was regarded as heterozygous. Finally, the genotype matrix was imputed using the Nearest Neighbor (KNN) algorithm with parameters of w = 70, *p* = 7, k = 7, and f = 0.8 [41]. Using three random test data, we estimated an accuracy rate of 96.4% with these same parameters.

### Population structure evaluation and LD analysis

The population structure of the tested population was determined using the principle component analysis (PCA) approach as incorporated in the software package GAPIT [42]. The calculation of LD between pairs of SNPs was performed using a combination of SNPs with MAFs > 0.04 and missing data < 10% and r^2^ (squared allele frequency correlations) using TASSEL version 3.0 [43]. In contrast to GWAS, missing SNP genotypes were not imputed with the major allele before LD analysis. Parameters in the program included MAF (≥ 0.04) and the integrity of each SNP (≥ 80%).

### Association mapping

The association signals were estimated based on 33,194 SNPs from 193 tested accessions. The Bonferroni method at α ≤ 0.05 (≤ 2.58 × 10^− 4^) was utilized to calculate the *p* value, which was set as the threshold to declare whether significant association signals existed [44].

### Definition and verification of candidate genes

Candidate genes located within the 100-kb LD segment near a peak SNP were identified. Biosynthesis of unsaturated FAs of soybean was used for FA-related gene screening (https://www.kegg.jp/). The expression level of each FA-related gene was determined by real-time reverse transcription polymerase chain reaction (RT-PCR) analyses. Real-time RT-PCR amplifications were performed using the real-time RT-PCR kit according to the manufacturer’s instructions (Takara, Japan) on CFX96 Touch™ Real-Time PCR Detection System (Bio-Rad, USA). About 1 μg of total RNA was used for each reverse transcription. Each amplification reaction was performed with 1 μL of the resulting cDNA first-strand cDNA synthesis solution, 0.2 μM of each primer, and 10 μL of SYBR Green PCR Master Mix, in a total reaction of 20 μL. The PCR cycling conditions were as follows: 95 °C for 5 s, 60 °C for 20 s, 72 °C for 20 s for 40 cycles and 60 °C for 1 min. The relative mRNA level of each candidate gene was evaluated against soybean actin4 (GmACTIN) (GenBank Accession Number AF049106) reference gene in three replicates. The sequences of the primer pairs used in amplifying the candidate genes are presented in Additional file 6: Table S3.

## Additional files

Additional file 1: **Table S1.** Basic genetic parameter statistics for unsaturated FA in the tested soybean population (*n* = 194). (XLSX 10 kb)

Additional file 2: Supplementary Data 1 Genotype data of association panel. (ZIP 1264 kb)

Additional file 3: **Table S2.** Primers for quantitative RT-PCR. (TIF 622 kb)

Additional file 4: **Table S3.** Genes in 100 kbp flanking regions of peak SNP associated with three unsaturated FAs of soybean. (XLSX 118 kb)

Additional file 5: **Figure S1.** Linkage disequilibrium pattern and population structure of the association panel. a: The linkage disequilibrium (LD) decay of the genome-wide association study (GWAS) population. b: The first three principal components of the 22,742 SNPs used in the GWAS. c: The population structure of the soybean germplasm collection reflected by the first 20 principal components. d: A heat map of the kinship matrix of the 194 soybean accessions calculated from the same 22,742 SNPs used in the GWAS. (TIF 126 kb)

Additional file 6: **Figure S2.** Functional categories of the genes in 100-kbp flanking regions around peak SNPs. (XLSX 8 kb)

## Abbreviations

Chr: Chromosomes
FA: unsaturated fatty acid
GO: Gene ontology
GWAS: Genome-wide association
LD: Linkage disequilibrium
LG: Linkage groups
LLA: Linoleic acid
LNA: Linolenic acid
MAF: Minor allele frequencies
MAS: Marker-assistant selection
OA: Oleic acid
PA: Palmitic acid
QTL: Quantitative trait loci
QTN: Quantitative trait nucleotide
SA: Stearic acid
SNP: Single Nucleotide Polymorphism
