# Supplementary figures and images for: Genome-wide association and transcriptional studies reveal novel genes for unsaturated fatty acid synthesis in a panel of soybean accessions
Source: BMC Genomics. 2019 Jan 21;20:68. doi: 10.1186/s12864-019-5449-z (PMC6341525; doi:10.1186/s12864-019-5449-z)

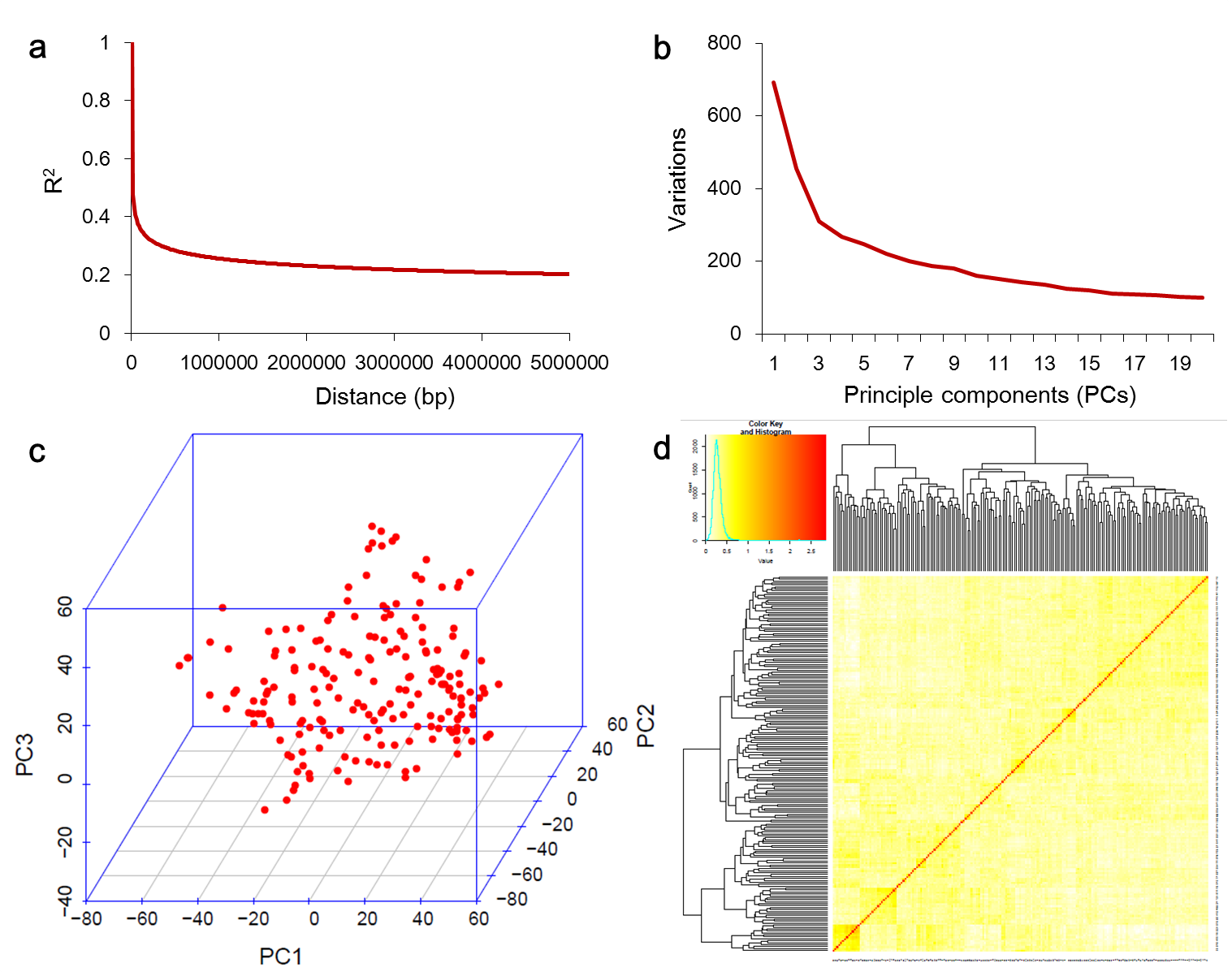

Supplement: Supplementary file 3 — Table S2. Primers for quantitative RT-PCR. (TIF 622 kb) [file 12864_2019_5449_MOESM3_ESM.tif]

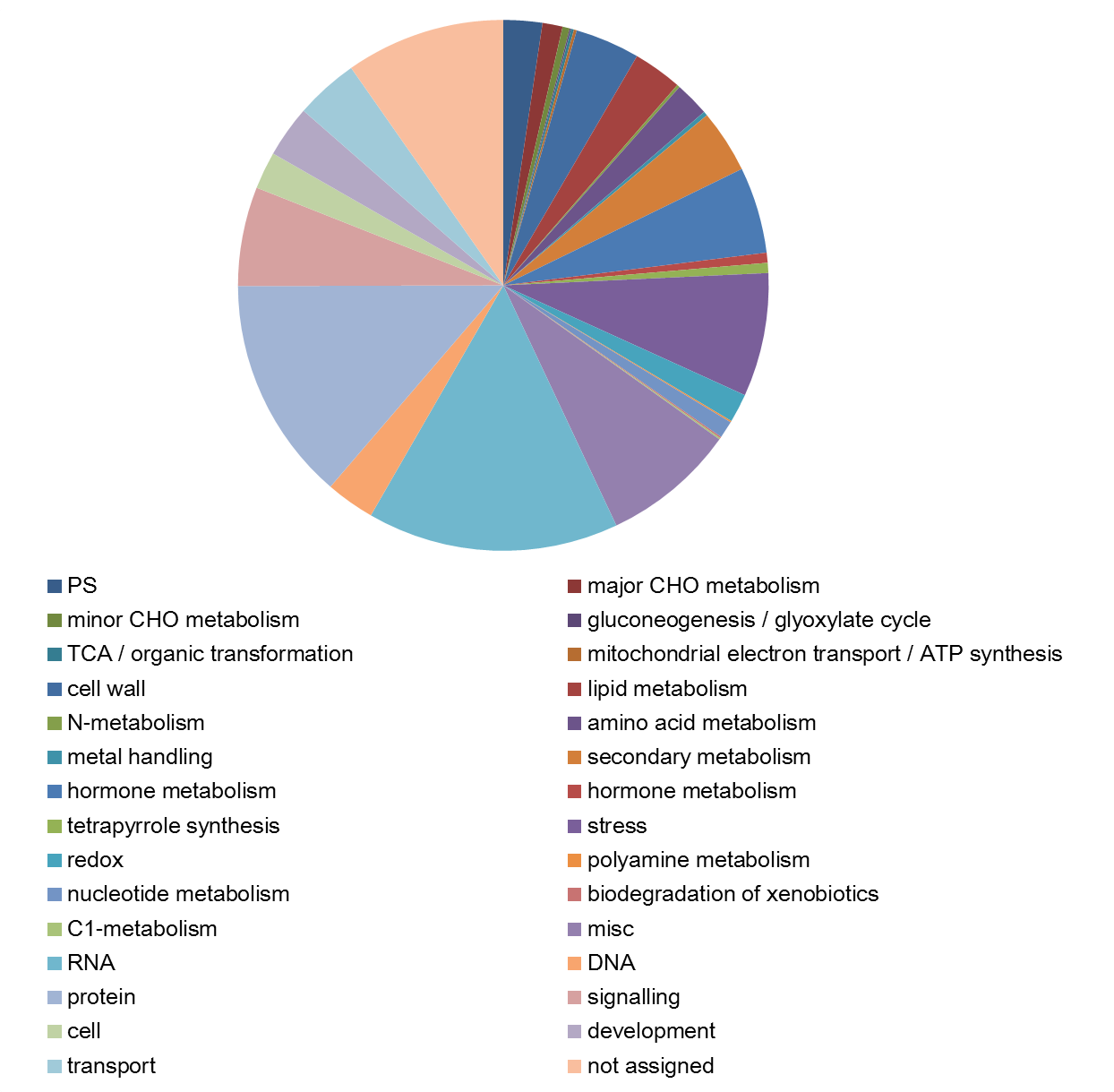

Supplement: Supplementary file 5 — Figure S1. Linkage disequilibrium pattern and population structure of the association panel. a: The linkage disequilibrium (LD) decay of the genome-wide association study (GWAS) population. b: The first three principal components of the 22,742 SNPs used in the GWAS. c: The population structure of the soybean germplasm collection reflected by the first 20 principal components. d: A heat map of the kinship matrix of the 194 soybean accessions calculated from the same 22,742 SNPs used in the GWAS. (TIF 126 kb) [file 12864_2019_5449_MOESM5_ESM.tif]
